# Efficacy and safety of perfluorohexyloctane (PFHO) in patients with dry eye disease (DED) due to meibomian gland dysfunction (MGD): Systematic review and meta-analysis

**DOI:** 10.1097/MD.0000000000043026

**Published:** 2025-07-18

**Authors:** Rabbia Munsab, Rabia Zafar, Anoosha Fatima, Javeria Taj, Muhammad Usman, Muhammad Umair Anjum, Muhammad Hasanain, Pratik Bhattarai

**Affiliations:** a Dow Medical College, Karachi, Pakistan; b Manipal College of Medical Sciences, Pokhara, Nepal.

**Keywords:** blurred vision, dry eye disease, eye dryness score, eye pain, meibomian gland dysfunction, perfluorohexyloctane, total corneal fluorescein staining

## Abstract

**Background:**

Dry eye disease (DED) associated with meibomian gland dysfunction lacks a conclusive treatment, placing existing solutions under the burden of side effects and limitations. This study seeks to assess the efficacy and safety of perfluorohexyloctane (PFHO) in treating DED associated with meibomian gland dysfunction.

**Methods:**

A thorough search encompassing Medline, Cochrane Central Register, and Google Scholar was conducted until September 15, 2023. Four randomized controlled trials were assessed for PFHO’s safety and efficacy. Studies were managed with EndNote and Excel for dual-phase screening by reviewers. Bias risk was evaluated with the risk of bias 2.0 Cochrane tool, and comparative outcomes were synthesized using RevMan software.

**Results:**

In this meta-analysis, we pooled data from 4 randomized controlled trials involving 1754 patients. Significant reductions were observed in total corneal fluorescein staining score mean difference = −0.87 (95% CI −1.06, −0.68, *P* < .00001), eye dryness score mean difference = −7.73 (95% CI −9.17, −6.29, *P* < .00001), and central Corneal Fluorescein Staining score risk ratios (RR) = 5.11 (95% CI: 1.75–14.93, *P* = .003). Subgroup analysis highlighted increasing differences over time, notably at week 8. Safety analysis showed decreased risk of eye pain RR = 0.70 (95% CI: 0.27, 1.80, *P* = .46) and blurred vision RR = 5.11 (95% CI: 1.75, 14.93, *P* = .003) with PFHO. There was no significant difference noted in serious adverse events RR = 1.38 (95% CI: 0.25, 7.60, *P* = .71), ocular TEAS RR = 1.05 (95% CI: 0.81, 1.37, *P* = .69) and non-ocular TEAS RR = 1.02 (95% CI: 0.75, 1.40, *P* = .89).

**Conclusion:**

PFHO marks a novel treatment for DED, targeting tear evaporation with its unique water-free, preservative-free preparation. It has proved to be highly effective in alleviating eye dryness and associated symptoms. While minor side effects are seen in a small subset of patients, the overall safety is promising. Further long-term and larger sample size studies will offer a more thorough understanding of this new drug.

## 1. Introduction

Dry eye disease (DED), also called keratoconjunctivitis sicca, is a widespread ocular condition that impacts millions of individuals globally, with prevalence estimates varying significantly, ranging from 5% to 50% in adults.^[1]^ Despite its common occurrence, DED poses a unique challenge in the field of ophthalmology as it remains one of the most frequently misdiagnosed and improperly treated conditions.^[2]^ The spectrum of DED symptoms spans from mild discomfort to incapacitating eye conditions, all of which significantly diminish an individual’s overall quality of life.^[3]^ The economic consequences of DED in the United States alone are staggering, with annual costs exceeding $55 billion due to reduced workplace productivity and increased work absences.^[4]^

One of the primary culprits behind DED is meibomian gland dysfunction (MGD). MGD is characterized by a reduction or alteration in meibum secretion, a crucial component responsible for maintaining the stability of the tear film’s lipid layer.^[5]^ The disruption of this lipid layer accelerates the evaporation of the tear film, setting in motion a relentless cycle of DED. This cycle includes tear film instability, desiccation, elevated tear hyperosmolarity, inflammation, and damage to the ocular cells.

Perfluorohexyloctane (PFHO), also known as Meibo is an eye drop formulated to manage DED effectively. It is an anhydrous semi-fluorinated alkane, free from water and preservatives, and acts as an amphiphilic molecule with lower surface tension than traditional alkanes.^[6]^ This property allows it to spread rapidly across the eye, forming a durable protective layer at the tear film-air interface that prevents the evaporation of the aqueous phase and reduces stress on the eyelids during blinking.^[7]^ With a refractive index similar to water and a small drop size of about 10 µL, it ensures prolonged residence time without impairing vision. Research shows that when applied over saline, PFHO reduces evaporation rates by approximately 80%, indicating its role in stabilizing the lipid layer of the tear film.^[8]^ By minimizing tear film evaporation, PFHO helps mitigate risks of drying and hyperosmolarity, thereby reducing inflammation and promoting healing of the ocular surface.^[9]^

DED has prompted a variety of therapeutic interventions, including artificial tear inducers, tear-stimulating medications, anti-inflammatory agents, and surgical options.^[10,11]^ Specifically, treatments such as omega-3 fatty acids, hyaluronic acids, tetracyclines, and secretagogues have been utilized, alongside artificial tear substitutes and anti-inflammatory eye drops.^[12]^ However, many of these options offer only temporary relief and fail to address the underlying causes of DED. For instance, high-viscosity tears and antibiotics provide limited benefits, and the efficacy of omega-3 supplements remains uncertain.^[13]^ Preservative-free tear supplements^[14]^ have proven less effective than their preservative-conjugated counterparts, while steroidal anti-inflammatory treatments carry risks of serious side effects, such as glaucoma and cataracts. Cyclosporine shows result when combined with artificial tears, but evidence of its impact on tear production and stability is inconsistent.^[15]^ Additionally, other treatments like Varenicline nasal spray and Lipiflow® are still under investigation, highlighting the need for effective and accessible options.^[16,17]^

Given these limitations in existing therapies, there is a significant gap in the treatment landscape for DED, particularly concerning MGD. This underscores the necessity for newer, more effective treatments like PFHO. Its unique mechanism of action involves forming a protective layer that stabilizes the tear film and minimizes evaporation, differentiating it from traditional therapies that do not address the root causes of dry eye symptoms. Therefore, our meta-analysis aims to evaluate the efficacy and safety of PFHO, establishing its potential as a viable treatment for DED and addressing the critical need for improved therapeutic options. The present analysis aims to consolidate the existing body of knowledge on PFHO in the context of DED. By meticulously examining all available data from randomized controlled trials (RCTs).^[8,18–20]^

## 2. Methods and materials

This systematic review and meta-analysis adhered to the rigorous methodologies outlined in the Cochrane Handbook for Systematic Reviews of Interventions^[21]^ and adhered to the reporting standards set forth in the Preferred Reporting Items for Systematic Reviews and Meta-Analysis (PRISMA) 2020 guidelines.^[21]^

### 2.1. Search strategy

For our study, we conducted a comprehensive search of the Medline and Cochrane Central Register of Controlled Trials databases, spanning from their inception to September 15, 2023. Our search was not restricted by date or language, ensuring inclusivity. In addition to these databases, we explored Grey Literature resources via Google Scholar. Our search strategy employed a combination of MeSH terms and relevant keywords, including “Perfluorohexyl-octane,” “Meibomian Gland Dysfunction,” and “Dry Eye Syndromes.” For the detailed search strategy, data is provided in Table 1.

**Table 1:** Search string and result of literature search using different databases.

Database	Search strategy	Results
MEDLINE	(perfluorhexyl-octan OR perfluorohexyloctane OR F6H8 cpd) AND (Meibomian Gland Dysfunction OR Dry Eye Syndrome OR Dry Eye Disease OR Evaporative Dry Eye Disease)	21
Google Scholar	(perfluorhexyl-octan OR perfluorohexyloctane OR F6H8 cpd) AND (Meibomian Gland Dysfunction OR Dry Eye Syndrome OR Dry Eye Disease OR Evaporative Dry Eye Disease)	3
Cochrane	(perfluorhexyl-octan OR perfluorohexyloctane OR F6H8 cpd) AND (Meibomian Gland Dysfunction OR Dry Eye Syndrome OR Dry Eye Disease OR Evaporative Dry Eye Disease)	2580

### 2.2. Literature search

**Figure 1.**
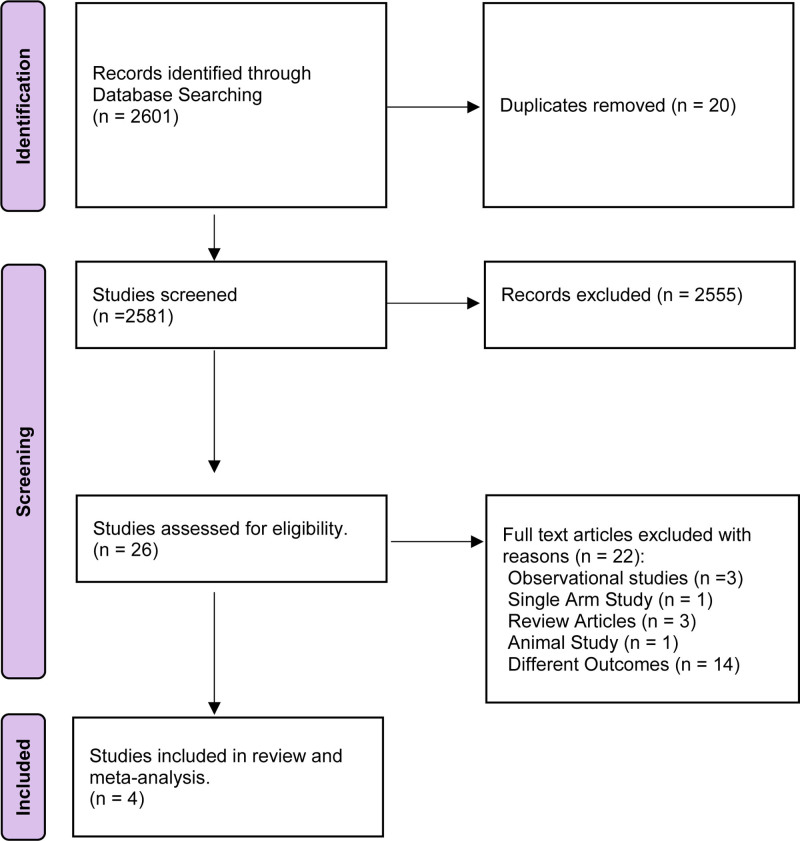
PRISMA 2020 flow diagram for new systematic reviews which included searches of databases and registers only. PRISMA = Preferred Reporting Items for Systematic Reviews and Meta-Analysis.

For the entire screening process, EndNote software v20 was utilized. In the first phase of screening, all the duplicates were detected by EndNote and removed. In the second phase of screening, 2 investigators (R.Z. and R.M.) independently reviewed all the literature by reading titles and abstracts to ensure their quality to be included in data extraction, and the remaining duplicates were removed manually. Disagreements were resolved with discussion or the consensus of the corresponding investigator (J.T.). Figure 1 shows the PRISMA flow diagram illustrating the study selection process.

### 2.3. Inclusion and exclusion criteria

Adult patients aged 18 years or older, with a self-reported history of DED in both eyes for at least 6 months, were eligible for the study if they met specific inclusion criteria in at least one eye at both screening and randomization. These criteria included tear film breakup time of 5 seconds or less, and unanesthetized Schirmer tear test I of 5 mm or more. A total MGD score of 3 or more, a total corneal fluorescein staining (tCFS) score ranging from 4 to 11 on the National Eye Institute scale, and an Ocular Surface Disease Index (OSDI) score of 25 or higher. Exclusion criteria encompassed clinically significant slit-lamp findings or abnormal lid anatomy, active blepharitis, active ocular allergies, ocular or periocular malignancy, recent intraocular or ocular laser surgery within 6 months, LipiFlow or similar meibomian gland procedures within the prior 6 months. Contact lens use within the previous month, recent topical medication use, history of isotretinoin use, ongoing ocular or systemic infection, uncontrolled systemic diseases, or a history of herpetic keratitis. Additionally, patients with poor best-corrected visual acuity in both eyes were excluded from participation. Other details are provided in Table 2.

**Table 2:** Baseline values of perfluorohexyloctane and placebo group among all included RCTs.

Study	Baseline	Perfluorohexyloctane	Placebo
Tauber 2023	Age (mean)	60.3	61.6
	tCFS score, study eye	6.7 (1.8)	6.7 (1.9)
	Eye dryness score	66.5 (19.1)	66.8 (18.7)
	Eye burning/stinging score	53.0 (26.7)	52.1 (26.6)
	Total MGD score	7.4 (3.1)	7.7 (3.2)
	TFBUT in the study eye, seconds	3.2 (0.8)	3.3 (0.8)
	Unanesthetized Schirmer, I test in the study eye	12.0 (8.3)	11.7 (7.6)
	OSDI score	53.9 (17.6)	54.4 (17.0)
	BCVA (logMAR)	0.07 (0.1)	0.09 (0.1)
Sheppard 2023	Age (mean)	53.3	53.8
	tCFS score, study eye	7.0 (2.0)	7.1 (2.1)
	Eye dryness score	64.7 (19.5)	64.3 (19.8)
	Eye burning/stinging score	50.1 (25.8)	48.4 (26.2)
	Total MGD score	7.9 (3.5)	8.1 (3.5)
	TFBUT in the study eye, seconds	3.2 (0.9)	3.1 (0.9)
	Unanesthetized Schirmer, I test in the study eye	12.7 (7.5)	12.8 (7.9)
	OSDI score	55.2 (17.4)	55.8 (17.2)
	BCVA (logMAR)	0.07 (0.1)	0.07 (0.1)
Tian 2023	Age mean (S.D)	45.4 (15.2)	43.7 (15.1)
	tCFS score	6.2 (1.9)	6.3 (1.7)
	Eye dryness score	64.7 (15.1)	65.6 (16.5)
	Schirmer, I test without anesthesia, mm at 5 min	12.9 (7.0)	13.2 (7.0)
	MGD score	8.4 (3.7)	8.4 (3.8)
	TFBUT	2.968 (0.896)	2.875 (0.913)
	OSDI score	55.8 (16.6)	56.2 (16.6)
Tauber 2021	Age (mean)	53.0	53.8
	tCFS score	7.0 (2.2)	6.7 (2.0)
	Eye dryness score	68.6 (21.8)	66.8 (21.7)
	Schirmer, I test	14.6 (8.9)	14.3 (8.8)
	MGD score	7.6 (3.5)	7.3 (3.4)
	TFBUT	3.0 (0.93)	3.0 (0.91)
	OSDI score	55.3 (17.4)	54.0 (18.9)

BCVA = best-corrected visual acuity, tCFS = tCFS = total corneal fluorescein staining, OSDI = Ocular Surface Disease Index, TFBUT = tear film break-up time.

### 2.4. Data extraction

In this current review, we meticulously extracted data from the included studies, encompassing data on study characteristics (authors, design, outcomes), patient population (participant count, age, baseline characteristics), intervention (dosage regimens), and outcomes comprising efficacy and safety measures. Our data extraction process was facilitated by Microsoft Excel 2019. Two authors, J.T. and A.F., extracted studies for inclusion based on the criteria outlined above, while discrepancies were resolved by the third author, M.U. All studies included in our analysis had complete data, and no missing data were identified. For categorical outcomes, we recorded the number of events and the total number of patients, while for continuous outcomes, we collected sample sizes, means, standard deviations, and standard errors.

### 2.5. Risk of bias and quality assessment

The methodological quality of the studies included in our analysis, as well as the assessment of the risk of bias, underwent an independent evaluation by 2 investigators, namely R.Z. and R.M. To conduct this evaluation, we employed the Cochrane “Risk of Bias” tool for randomized trials, known as risk of bias (ROB) 2.0.^[22]^ ROB 2.0 scrutinizes bias across 5 critical domains: (1) bias stemming from the randomization process; (2) bias attributable to deviations from intended interventions; (3) bias due to missing outcome data; (4) bias in the measurement of the outcome; and (5) bias in the selection of the reported results. Each study included in our analysis underwent assessment using this tool, and the source of bias was categorized as high, low, or unclear, consequently determining the overall risk of bias as high, low, or having some concerns. In instances where disagreements arose during the assessment, a third author, J.T., intervened to facilitate resolution.

### 2.6. Primary and secondary outcomes

The main outcomes of the study included efficacy and safety measures. Efficacy encompassed tCFS, eye dryness score, central Corneal Fluorescein Staining (cCFS), eye burning/stinging score, and the OSDI score. Staining was assessed through the National Eye Institute scale, whereas eye burning/stinging and eye dryness symptoms were assessed using a visual analog scale while the OSDI score was assessed using the OSDI questionnaire.

Safety assessments covered blurred vision, eye pain, treatment-emergent adverse events (TEAEs), treatment-related adverse events (TRAEs), serious adverse events (SAEs), and treatment discontinuation. Safety evaluations included visual acuity, biomicroscopy, intraocular pressure, and fundoscopy.

TEAEs comprised new-onset health issues during the trial and exacerbations of preexisting conditions. Any adverse events (AEs) that were, possibly, or undetermined in association with the study drugs were designated as TRAEs.

### 2.7. Data analysis

Data analysis was conducted utilizing RevMan (Review Manager) version 5.3, as endorsed by the Cochrane Collaboration.^[23]^ Our meta-analysis was executed employing the random-effects model, with statistical significance set at *P* < .05 and a 95% confidence level. To assess statistical heterogeneity, we employed the I2 statistic. Continuous variables, such as tCFS score and cCFS score, were analyzed using the mean difference and subsequently pooled using the Generic Variance method. For dichotomous outcomes like SAEs and AEs, risk ratios (RR) were employed and pooled using inverse variance weighting. Additionally, we performed subgroup analysis by categorizing efficacy measurements into different time points, specifically at the 2nd, 4th, and 8th weeks, to provide more comprehensive insights.

## 3. Results

**Figure 2.**
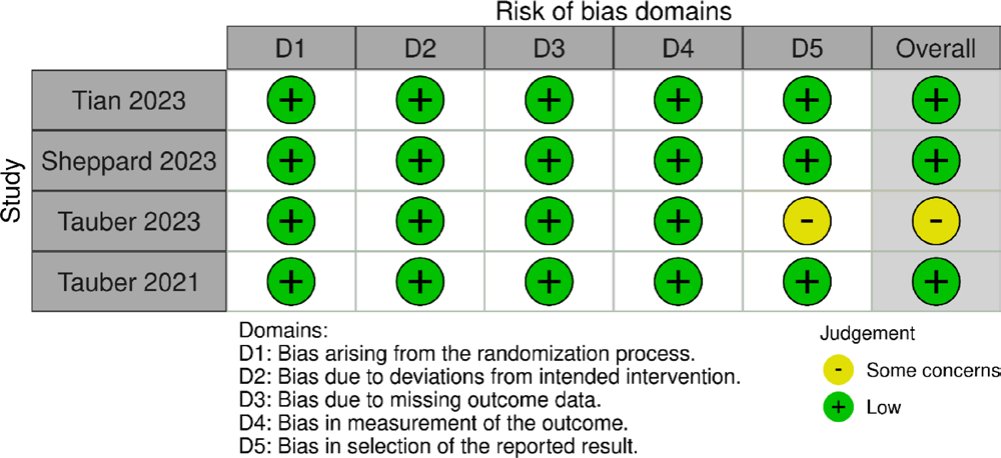
Quality assessment using ROB 2.0 Cochrane risk of bias tool. ROB = risk of bias.

The initial search resulted in 2601 studies, and after deleting duplicates, 2581 studies were left. Upon reviewing the titles and abstracts, 2555 studies did not meet our inclusion criteria and were excluded. Subsequently, 26 full-text papers were thoroughly examined for eligibility. Our meta-analysis eventually included 4 RCTs that met our predefined criteria for inclusion. Additional screening process details are outlined in the PRISMA flowchart (Fig. 1). All included studies were evaluated as having a low risk of bias using the ROB 2 Cochrane risk of bias tool for RCTs except for moderate bias found in Tauber 2023 (Fig. 2).

### 3.1. Study characteristics and demographic features

This comprehensive analysis encompasses data from 4 randomized double-blind, placebo-controlled trials, comprising a total of 1754 patients. All the trials assessed in this study employed a parallel design. The mean age within the PFHO group was 55.25 years, while in the placebo group, it was 53.47 years. Details of study characteristics and baseline values are provided in Tables 2 and 3.

**Table 3:** Study characteristics of all included RCTs.

Study characteristics	Tian 2023	Sheppard 2023	Tauber 2023	Tauber 2021
1. Country	China	USA	USA	USA
2. Study type	RCT	RCT	RCT	RCT
3. Number of patients				
Perfluorohexyloctane	156	309	303	114
Placebo	156	311	294	111
4. Amount of dose				
Perfluorohexyloctane	N/A	1 drop	1 drop	1 drop
Placebo	N/A	N/A	1 drop normal saline	1 drop normal saline
5. Duration:				
Perfluorohexyloctane	4 times/day	4 times/day	4 times/day	4 times/day
Placebo	4 times/day	4 times/day	4 times/day	4 times/day
6. Route of administration				
Perfluorohexyloctane	eye drops	eye drops	eye drops	eye drops
Placebo	eye drops	eye drops	eye drops	eye drops
7. Follow Up:				
Perfluorohexyloctane	57 days	8 weeks	8 weeks	8 weeks
Placebo	57 days	8 weeks	8 weeks	8 weeks
8. Inclusion criteria	1.Participants aged 18 years or older. 2.Complaint of DED symptoms for 6 months or longer at screening 3.OSDI score of 25 or higher 4.tear film breakup time (TFBUT) of 5 seconds or less 5.Schirmer I test without anesthesia of 5 mm or more at 5 minutes, 6.tCFS score of 4 to 11 on the National Eye Institute scale. 7.MGD score of 3 or more.	1.Participants aged 18 years or older. 2.Complaint of DED symptoms for 6 months or longer at screening. 3.OSDI score of 25 or higher. 4.Tear film breakup time (TFBUT) of 5 seconds or less. 5.Schirmer I test without anesthesia of 5 mm or more in 5 minutes. 6.tCFS score of 4 to 11 on the National Eye Institute scale. 7.MGD score of 3 or more based on evaluation of 5 central meibomian glands on the lower eyelid, each scored from 0-3	1.Participants aged 18 years or older. 2. Self-reported DED symptoms for ≥ 6 months at screening. 3. OSDI score ≥ 25. 4. TFBUT ≤ 5 seconds. 5. Schirmer I test (without anesthesia) ≥ 5 mm at 5 minutes. 6.tCFS score of 4 to 11 (National Eye Institute scale). 7.MGD score of 3 or more, based on evaluation of 5 central meibomian glands on the lower eyelid, each scored from 0–3.	1. Participants aged ≥ 18 years. 2.History of DED in both eyes. 3.Main inclusion criteria for 1 eye (same eye) at screening and randomization: 4. TFBUT ≤ 5 seconds. 5. Schirmer I test ≥ 5 mm. 6. MGD score ≥ 3. 7.tCFS score between 4 and 11. 8. OSDI score ≥ 25.
9. Exclusion criteria	1.Slit-lamp findings or abnormal lid anatomy (e.g., trauma, pterygium, blepharitis). 2.Severe systemic autoimmune diseases. 3.Recent lacrimal plug placement/removal within 3 months. 4.DED due to scarring, radiation, burns, or malignancy. 5.Ocular/periocular malignancy. 6.Active ocular allergies or allergies to study drug/components. 7.Active infection. 8.Recent intraocular/ocular laser surgery within 6 months. 9.Uncontrolled systemic disease or herpetic keratitis history	1.Significant slit-lamp findings. 2.Active blepharitis or ocular allergies. 3.Recent eye surgeries or procedures within 6 months. 4.Recent contact lens use within 1 5.month. 6.Recent use of specific eye medications within 60 days. 7.History of isotretinoin use	1.Significant slit-lamp findings or lid issues. 2.DED due to scarring or malignancy. 3.Active ocular issues or infections. 4.Recent eye procedures in the past 6 months. 5.Recent contact lens use in the past month. 6.Recent use of certain eye medications in the past 60 days. 7.Poor best-corrected visual acuity in both eyes. 8.Continuous treatment period starting 1 day before baseline.	1.Slit-lamp findings or abnormal lid anatomy (e.g., trauma, pterygium, blepharitis). 2.Severe systemic autoimmune diseases. 3.Recent lacrimal plug placement/removal within 3 months. 4.DED due to scarring, radiation, burns, or malignancy. 5.Ocular/periocular malignancy. 6.Active ocular allergies or allergies to study drug/components. 7.Active infection. 8.Recent intraocular/ocular laser surgery within 6 months. 9.Uncontrolled systemic disease or herpetic keratitis history.
10.Efficacy outcome	tCFS score using the National Eye Institute scale (range, 0–15, with higher scores representing a worse condition), TFBUT, MGD score, and Schirmer I test	Corneal fluorescein staining assessed by the investigator in 5 corneal areas using the NEI scale (graded from 0 to 3, with a maximum total score of 15). Patient-reported symptom severity, including eye dryness and burning/stinging, rated using a VAS from 0 (no discomfort) to 100 (maximal discomfort)	Corneal fluorescein staining assessed by the investigator in 5 corneal areas using the NEI scale (graded from 0 to 3, with a maximum total score of 15). Patient-reported symptom severity, including eye dryness and burning/stinging, rated using a VAS from 0 (no discomfort) to 100 (maximal discomfort)	tCFS (NEI scale 0-3 for each of 5 cornea areas). Symptoms assessed with VAS (0–100 scale) for various discomforts. Frequency and awareness of dryness symptoms (VAS 0–100). OSDI questionnaire (total score 0–100, higher scores indicating worse condition). CFS subregions (NEI scale), conjunctival staining (Oxford scale), unanesthetized Schirmer I test, TFBUT, and meibomian glands evaluation.
11.Safety outcome	Safety assessments included intraocular pressure measurement and fundus photography at screening and day 57, visual acuity and slit lamp examination at each visit, and TEAEs	Safety assessments included AEs, best corrected visual acuity, slit lamp biomicroscopy, intraocular pressure, and dilated fundoscopy	Safety assessments included AEs, BCVA, slit lamp biomicroscopy, intraocular pressure, and dilated fundoscopy	Safety assessments included TEAEs and the following ophthalmic assessments: visual acuity, slit lamp biomicroscopy, intraocular pressure, and dilated fundoscopy

AEs = adverse events, BCVA = best-corrected visual acuity, NEI = National Eye Institute, OSDI = Ocular Surface Disease Index, TEAEs = treatment-emergent adverse events, TFBUT = tear film break-up time.

### 3.2. Outcomes

#### 3.2.1. Efficacy

**Figure 3.**
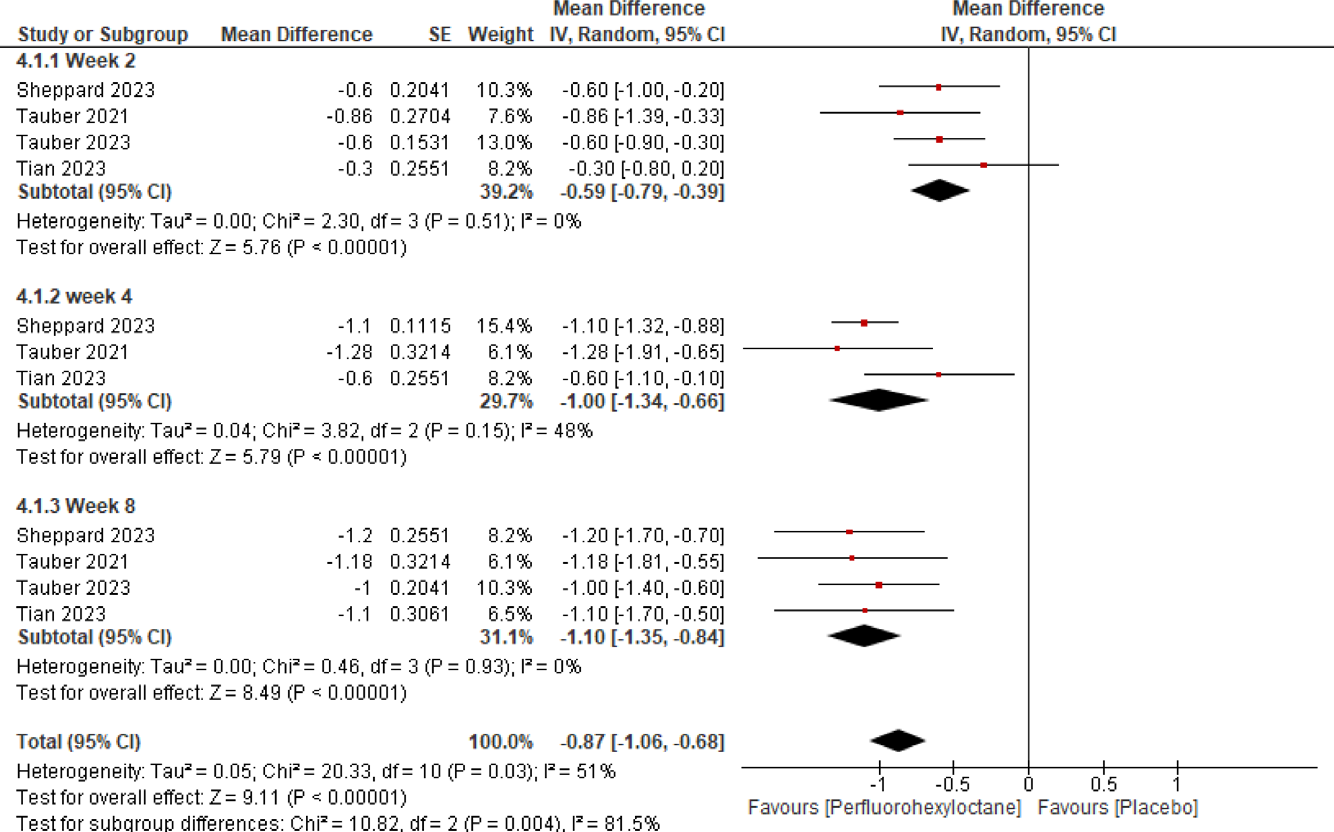
Assessment of efficacy of perfluorohexyloctane using tCFS score. tCFS = total corneal fluorescein staining.

**Figure 4.**
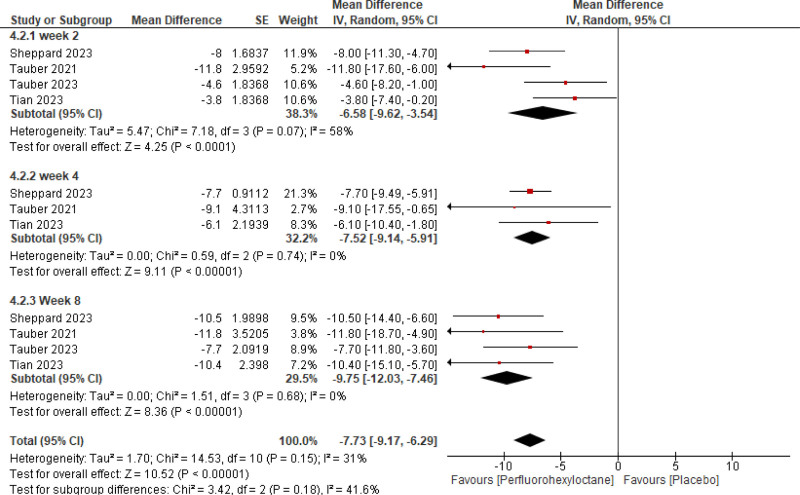
Assessment of efficacy of perfluorohexyloctane using eye dryness score.

**Figure 5.**
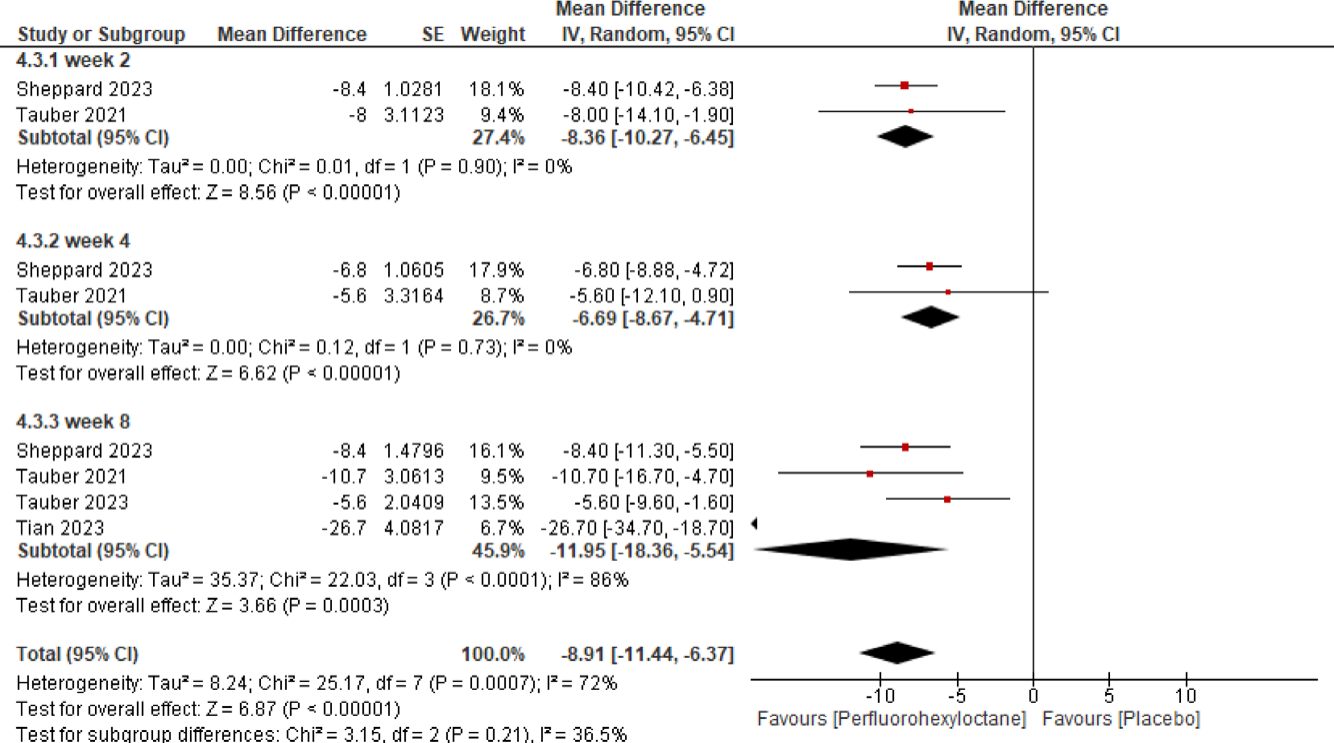
Assessment of efficacy of perfluorohexyloctane using eye burning/stinging score.

**Figure 6.**
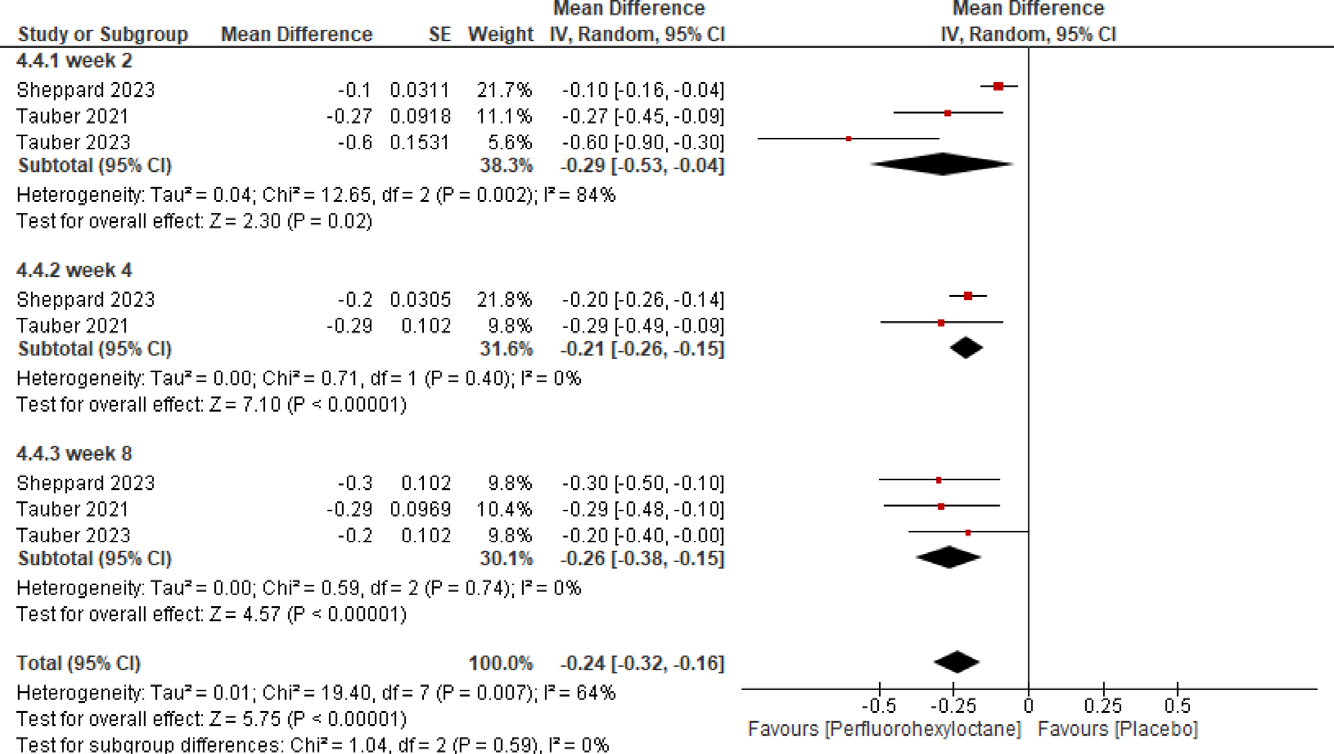
Assessment of efficacy of perfluorohexyloctane using cCFS score. cCFS = central corneal fluorescein staining

**Figure 7.**
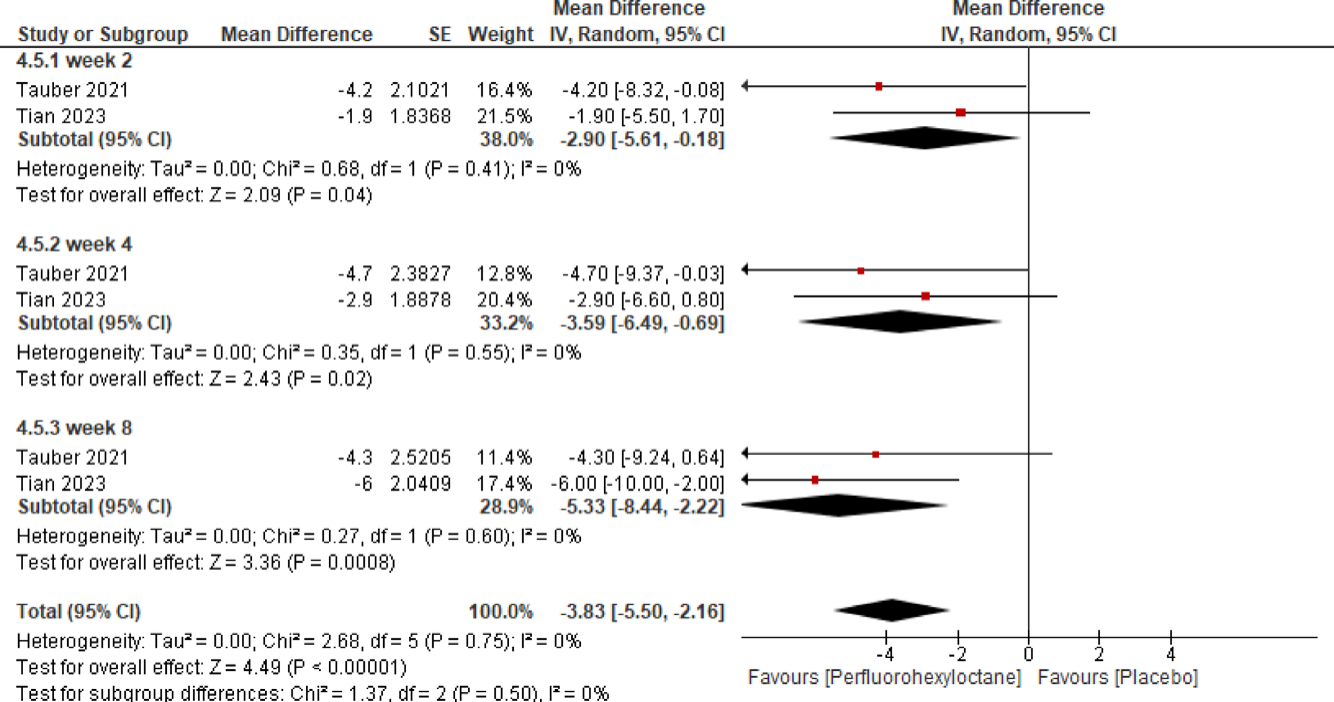
Assessment of efficacy of perfluorohexyloctane using OSDI score. OSDI = Ocular Surface Disease Index.

Pooled analysis of the included studies showed that PFHO significantly reduced tCFS score (mean difference [MD] = −0.87 [95% CI −1.06, −0.68, *P* < .00001], *I*^2^ = 51%) (Fig. 3), eye dryness score (MD = −7.73 [95% CI −9.17, −6.29, *P* < .00001], *I*^2^ = 31%) (Fig. 4), mcCFS score (MD = −0.24 [95% CI −0.32, −0.16, *P* < .00001], *I*^2^ = 64%) (Fig. 5), eye burning/stinging score (MD = −8.91 [95% CI −11.44, −6.37, *P* < .00001], *I*^2^ = 72%) (Fig. 6), and OSDI score (MD = −3.83 [95% CI −5.50, −2.16, *P* < .00001], *I*^2^ = 0%) (Fig. 7).

Stratified analysis was meticulously conducted to assess the temporal impact of both interventions at the 2nd, 4th, and 8th weeks. It reported a significant reduction across all scores assessed for measuring efficacy consistently at 2nd, 4th, and 8th week follow-up.

#### 3.2.2. Leave-1-out sensitivity analysis

**Figure 8.**
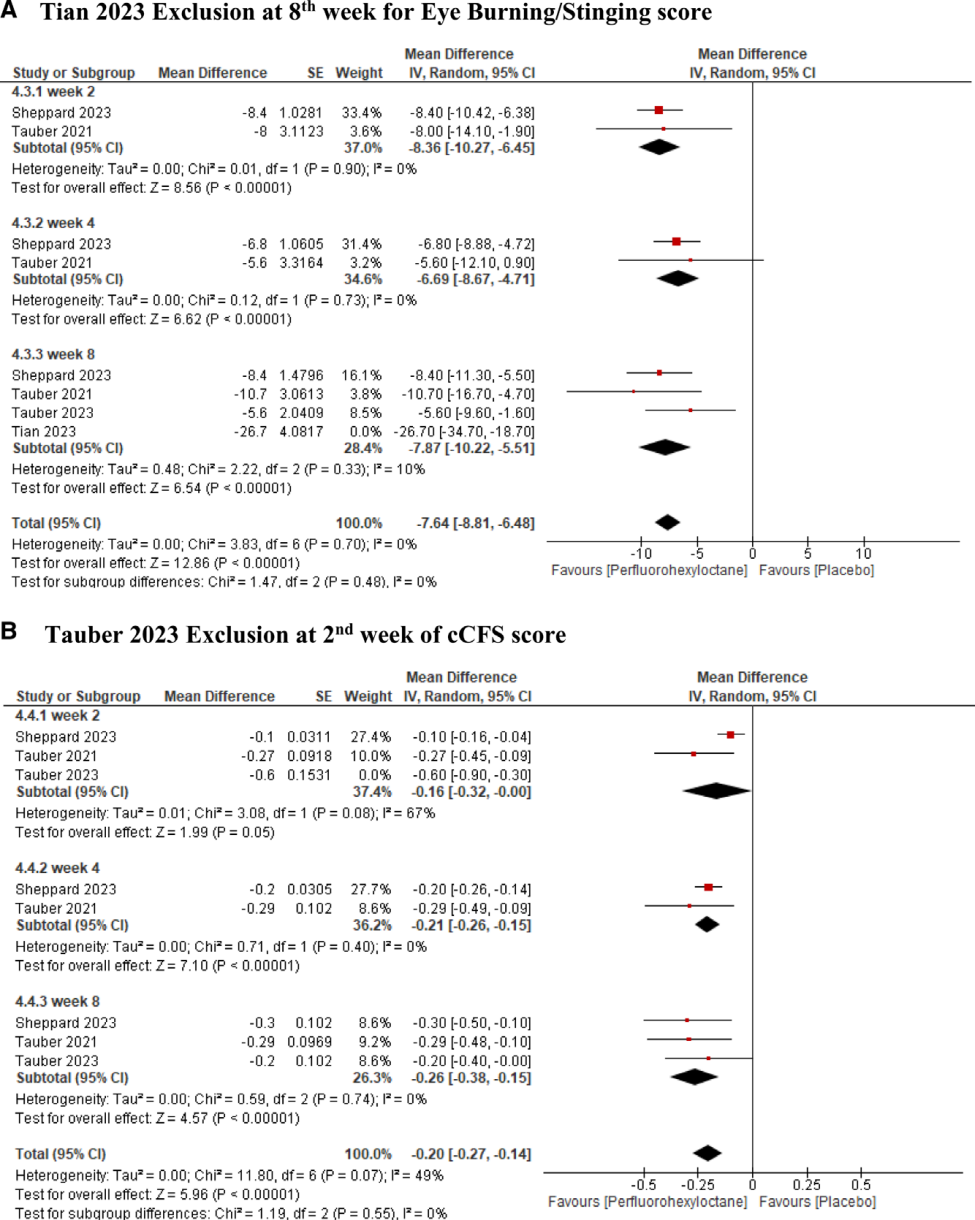
Result of leave one out analysis. (A) Tian 2023 exclusion at 8th week for eye burning/stinging score. (B) Tauber 2023 exclusion at 2nd week of cCFS score. cCFS = central corneal fluorescein staining.

Leave-one-out analysis for Eye Burning Score at 8th week showed that after removal of Tian 2023 study,^[9]^ the mean difference changed significantly from −11.95 (95% CI −18.36, 5.54) to −7.87 (95% CI −10.22, −5.51), and the sensitivity analysis showed a significant decrease in heterogeneity from (*I*^2^ = 86%, *P* < .0001) down to (*I*^2^ = 10%, *P* = .00001) on its removal (Fig. 8A).

Leave-one-out analysis for cCFS score at 2nd week showed that after removal of Tauber 2023 study,^[11]^ the mean difference changed slightly from −0.29 (95% CI −0.53, 0.04) to −0.16 (95% CI −0.32, −0.00), and the sensitivity analysis showed a slight decrease in heterogeneity from (*I*^2^ = 84%, *P* < .002) down to (*I*^2^ = 67%, *P* = .08) on its removal (Fig. 8B).

### 3.3. Safety

#### 3.3.1. Eye pain and blurred vision

**Figure 9.**
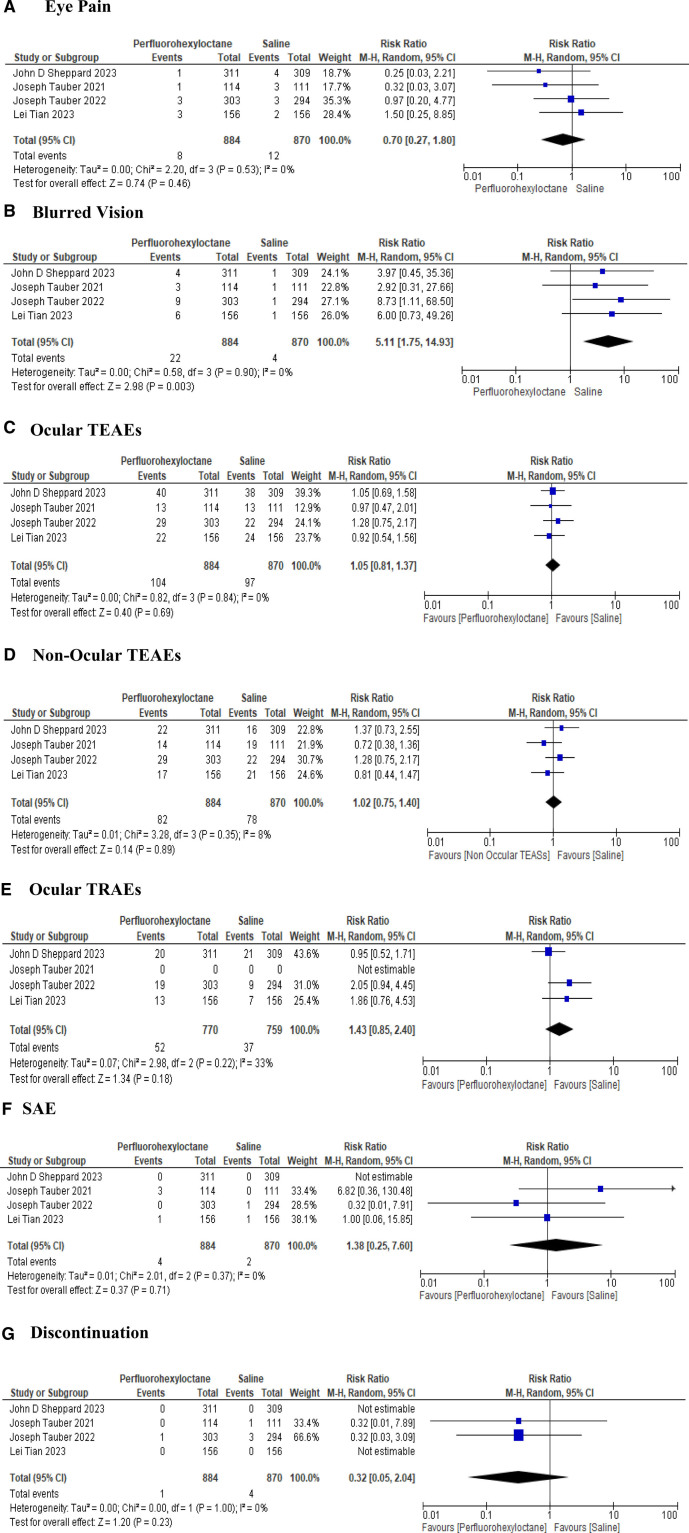
Forest plots for safety assessment of perfluorohexyloctane. (A) Eye pain. (B) Blurred vision. (C) Ocular TEAEs. (D) Non-ocular TEAEs. (E) Ocular TRAEs. (F) SAE. (G) Discontinuation. TEAEs = treatment-emergent adverse events.

PFHO was associated with a low risk of eye pain (RR 0.70 [95% CI: 0.27, 1.80], *P* = .46), while an increased risk of blurred vision (RR 5.11 [95% CI: 1.75, 14.93], *P* = .003) with no significant heterogeneity (*I*² = 0%) as compared to placebo (Fig. 9A and B).

#### 3.3.2. Ocular and non-ocular TEAEs

Our analysis reported no significant difference in the incidence of both ocular TEAEs (RR 1.05 [95% CI: 0.81, 1.37], *P* = .69, *I*² = 0%) and non-ocular TEAEs (RR 1.02 [95% CI: 0.75, 1.40], *P* = .89, *I*² = 8%) when comparing PFHO with the control group (Fig. 9C and D).

#### 3.3.3. Ocular and non-ocular TRAEs

No significant difference was recorded in either group for the presence of ocular TRAEs (RR 1.43 [95% CI: 0.85, 2.40], *P* = .18, *I*² = 33%) (Fig. 9E). Notably, non-ocular TRAEs could not be estimated due to limited data. Remarkably, none of the participants experienced any non-ocular TRAEs.

#### 3.3.4. Serious adverse events SAEs

Regarding SAEs, the analysis revealed a relative risk of RR = 1.38 (95% CI: 0.25, 7.60, *P* = .71) with no significant heterogeneity among the studies (*I*² = 0%) (Fig. 9F).

#### 3.3.5. Treatment discontinuation

There were fewer treatment discontinuations in the PFHO group (RR = 0.32 [95% CI: 0.05, 2.04, *P* = .23]) in contrast to placebo. However, results were insignificant with no significant heterogeneity (*I*² = 0%) (Fig. 9G).

For summary of all the outcomes refer to Tables 4 and 5.

**Table 4:** Summary of quantitative analysis and heterogeneity analysis.

Outcomes	Subgroups	Quantitative data synthesis				
		MD	95% CI	Z-value	*P*-value	
tCSF score	Week 2	‐0.59	‐0.79, −0.39	5.76		<.00001
	Week 4	‐1.00	‐1.34, −0.66	5.79		<.00001
	Week 8	‐1.10	‐1.35, −0.84	8.49		<.00001
Eye dryness score	Week 2	‐6.58	‐9.62, −3.54	4.25		<.00001
	Week 4	‐7.52	‐9.14, −5.91	9.11		<.00001
	Week 8	‐9.75	‐12.03, −7.46	8.36		<.00001
Eye burning/stinging score	Week 2	‐8.36	‐10.27, −6.45	8.56		<.00001
	Week 4	‐6.69	‐8.67, −4.71	6.62		<.00001
	Week 8	‐11.95	‐18.36, −5.54	3.66		.0003
cCSF score	Week 2	‐0.10	‐0.53, −0.04	2.30		.022
	Week 4	‐0.20	‐0.26, −0.15	7.10		<.00001
	Week 8	‐0.20	‐0.38, −0.15	4.57		<.00001
OSDI score	Week 2	‐2.90	‐5.61, −0.18	2.09		.04
	Week 4	‐3.59	‐6.49, −0.69	2.43		.02
	Week 8	‐3.53	‐5.44, −2.22	3.36		.0008

OSDI = Ocular Surface Disease Index.

**Table 5:** Summary of safety outcomes.

Outcomes	Quantitative data synthesis			
	RR	95% CI	Z-value	*P*-value
Eye pain	0.70	0.27, 1.80	0.74	.46
Blurred vision	5.11	1.75, 14.93	2.98	.003
Ocular TEAEs	1.05	0.81, 1.37	0.40	.69
Non-ocular TEAEs	1.02	0.75, 1.40	0.14	.89
Ocular TRAEs	1.43	0.85, 2.40	1.34	.18
SAEs	1.38	0.25, 7.60	0.37	.71
Treatment discontinuations	0.32	0.05, 2.04	1.20	.23

SAEs = serious adverse events, TEAEs = treatment-emergent adverse events.

## 4. Discussion

The meta-analysis consistently shows that PFHO is more effective than the placebo in improving eye dryness and related symptoms, boosting confidence in the findings. Beyond statistical significance, the results indicate a significant practical benefit for patients, particularly in the eye dryness score and OSDI score. Moreover, the analysis by week highlights variations in treatment efficacy timing, such as the tCFS score improving most at week 8 and the eye burning/stinging score showing the greatest improvement at week 2. These findings underscore the importance of personalized care tailored to specific symptoms and their progression over time.

In our comprehensive safety analysis comparing PFHO to placebo, several key findings emerged. A low risk of eye pain was noted in the PFHO group in contrast with the placebo. The same result is shown by the KALAHAR study with 1 % eye pain occurrence in overall patients as compared to a higher occurrence of 4% with lifitegrast. While blurred vision occurred more frequently with the use of PFHO, it is still a rare event with a low occurrence rate of 2.5% overall. Blurred vision is a common side effect with many DED treating eye drops, including high-viscosity eye drops and lifitegrast (Xiidra) with a 4.9% occurrence rate.^[24]^ The recent KALAHARI study reported a 1.4% occurrence of blurred vision, an even lower incidence with PFHO’s use.^[25]^ Therefore, it is not a major concern regarding PFHO’s overall safety profile.

It is worth noting that no significant differences were observed in ocular or non-ocular TEAEs. However, it is essential to emphasize that TRAEs did show a trend toward higher risk, although this trend did not reach statistical significance. This suggests a need for cautious monitoring when using PFHO clinically, particularly in terms of potential ocular AEs.

On a positive note, our analysis did not reveal significant differences in serious AEs, treatment discontinuation, or non-ocular TRAEs. These findings, coupled with the observed trends in ocular TRAEs, imply that while PFHO may offer clinical benefits in addressing eye dryness and associated symptoms, careful consideration of potential ocular side effects is warranted in its clinical application.

Furthermore, the absence of significant heterogeneity in various safety outcomes suggests consistency in the observed effects across the analyzed studies. However, it is important to recognize the limitations of our analysis, given the potential for larger studies to provide a more comprehensive understanding of PFHO’s safety profile.

DED stands as a widespread but often underdiagnosed and inadequately treated condition within the realm of ophthalmology. A wide variety of treatment options are available for DED, each targeting different pathophysiologic pathways. These include tear-film stabilizing hydroxypropyl cellulose ophthalmic insert (Lacrisert®),^[26]^ nicotinic acetylcholine receptor agonist varenicline nasal spray (Tyrvaya®)^[27]^ increasing basal tear production, immunosuppressive calcineurin inhibitor cyclosporine eye drops (Restasis®, Cequa®, Vevye®),^[28–30]^ lymphocyte function-associated antigen-1 antagonist lifitegrast ophthalmic solution (Xiidra®)^[24]^ inhibiting the interaction of lymphocyte function-associated antigen-1 cognate ligand intercellular adhesion molecule-1 further reducing inflammation, and corticosteroid loteprednol etabonate ophthalmic suspension (Eysuvis®) inhibiting inflammatory pathways.^[31]^ However, the recent single-ingredient, preservative-free prescription eye drop, PFHO, has been developed to target excessive evaporation.

Preclinical studies suggest that on instillation of PFHO in DED patients, it supplements the tear film lipid layer, spreads rapidly to form an anti-evaporative layer, and remains on the eye for at least 6 hours without blocking the diffusion of oxygen to the corneal surface. These results highlight the potential of PFHO to stabilize the tear film, reduce friction, and promote ocular surface healing.^[32]^

A multitude of treatments, ranging from omega-3 fatty acids and hyaluronic acids to tetracyclines and secretagogues, are employed, including artificial tear substitutes and anti-inflammatory eye drops.^[12]^ These treatments provide temporary relief but fail to address the underlying cause of DED. High-viscosity tears and antibiotics offer limited benefits, while omega-3 supplements’ efficacy remains uncertain.^[13]^ Preservative-free tear supplementation faced challenges and proved less effective than preservative-conjugated alternatives.^[14]^ Steroidal anti-inflammatory treatments showed success but came with the risk of side effects such as glaucoma, cataracts, and other complications. Cyclosporine demonstrated substantial efficacy, especially when combined with artificial tears.^[15]^ A Cochrane review provided low-certainty evidence that combining cyclosporine with artificial tears may reduce dry eye symptoms more effectively than artificial tears alone. However, evidence of cyclosporine’s impact on tear production and stability was inconsistent.^[33]^

Antibiotics displayed short-term advantages for MGD but lacked evidence of sustained improvement. Varenicline nasal spray offers a prospective treatment, although further data are needed.^[16]^ Intense pulsed light has shown efficacy in MGD-related DED, yet cost barriers remain, necessitating solutions for wider accessibility. Lipiflow® treatment appears promising but requires careful consideration due to study limitations.^[34]^ Considering these challenges, there is a pressing need for safe, affordable, and effective DED treatments that specifically target the signs and symptoms associated with MGD.

Our meta-analysis is noteworthy as it is the first to comprehensively assess the safety and efficacy of PFHO in addressing eye dryness and related symptoms, further emphasizing its unique contribution to the existing literature on this topic.

## 5. Limitations

The meta-analysis primarily focuses on short-term outcomes, specifically at weeks 2, 4, and 8, providing valuable insights into PFHO’s immediate effects on eye dryness and related symptoms. However, it is crucial to recognize the lack of comprehensive long-term data regarding its safety and efficacy. Acknowledging this limitation, future research should prioritize longer follow-up periods and posttreatment assessments to determine whether the observed short-term advantages are maintained over time and to identify any potential delayed adverse effects. This approach will provide a more thorough understanding of the drug’s long-term impact on patients with DED. None of the studies included in our meta-analysis specified autoimmune conditions, such as rheumatoid arthritis, in their inclusion or exclusion criteria. This limitation may impact the generalizability of our findings to populations with underlying autoimmune disorders. Furthermore, it is worth acknowledging that the presence of heterogeneity in some of our analyses suggests variability between the included studies. This heterogeneity could stem from differences in study populations, intervention protocols, or other uncontrolled factors that may influence the outcomes. Additionally, it is important to mention that our meta-analysis was based on a limited dataset, comprising only 4 RCTs. Data from the study by Tauber et al in 2023 was missing at the 4th-week time point which could have influenced the overall findings. Despite these limitations, our analysis provides valuable initial insights into the efficacy and safety of PFHO in the short term, highlighting the need for more extensive and longer-term investigations in this area.

## 6. Conclusion

PFHO marks a significant advancement in the management of DED, a prevalent ocular surface disorder affecting millions. By specifically targeting tear evaporation (one of the primary contributors to DED) PFHO offers a unique, first-of-its-kind, water-free, preservative-free treatment option. It has proved to be highly effective in alleviating eye dryness and associated symptoms. While some minor side effects have been reported in a small subset of patients, the overall safety profile remains robust. Highlighting the need for long-term studies with larger sample sizes will provide a more comprehensive understanding of its pharmacokinetics. The findings from this study underscore the clinical relevance of PFHO as an effective therapy for DED, ultimately paving the way for better management strategies and improving the quality of life for affected patients.

## Author contributions

**Conceptualization:** Muhammad Umair Anjum, Pratik Bhattarai.

**Data curation:** Anoosha Fatima, Muhammad Umair Anjum, Pratik Bhattarai.

**Formal analysis:** Pratik Bhattarai.

**Investigation:** Rabbia Munsab, Rabia Zafar, Anoosha Fatima, Javeria Taj, Muhammad Umair Anjum.

**Methodology:** Rabbia Munsab, Rabia Zafar, Anoosha Fatima, Muhammad Umair Anjum.

**Project administration:** Rabbia Munsab, Javeria Taj, Muhammad Hasanain, Pratik Bhattarai.

**Resources:** Anoosha Fatima.

**Supervision:** Anoosha Fatima, Muhammad Usman, Muhammad Umair Anjum, Muhammad Hasanain.

**Validation:** Muhammad Usman, Muhammad Hasanain.

**Visualization:** Muhammad Hasanain, Pratik Bhattarai.

**Writing – original draft:** Rabbia Munsab, Rabia Zafar, Javeria Taj.

**Writing – review & editing:** Rabia Zafar, Muhammad Usman.

## References

[1] Vernhardsdottir RR Magno MS Hynnekleiv L. Antibiotic treatment for DED related to meibomian gland dysfunction and blepharitis – a review. Ocul Surf. 2022;26:211–21. 36210626 10.1016/j.jtos.2022.08.010

[2] Bartlett JD Keith MS Sudharshan L Snedecor SJ. Associations between signs and symptoms of DED: a systematic review. Clin Ophthalmol. 2015;9:1719–30. 26396495 10.2147/OPTH.S89700 PMC4577273

[3] Rouen PA White ML. DED: prevalence, assessment, and management. Home Healthc Now. 2018;36:74–83. 29498987 10.1097/NHH.0000000000000652

[4] Yu J Asche CV Fairchild CJ. The economic burden of DED in the United States: a decision tree analysis. Cornea. 2011;30:379–87. 21045640 10.1097/ICO.0b013e3181f7f363

[5] Craig JP Nichols KK Akpek EK. TFOS DEWS II definition and classification report. Ocul Surf. 2017;15:276–83. 28736335 10.1016/j.jtos.2017.05.008

[6] Vittitow J Kissling R DeCory H Borchman D. In vitro inhibition of evaporation with PFHO, an eye drop for DED. Curr Ther Res Clin Exp. 2023;98. 10.1016/j.curtheres.2023.100704 PMC10300294 37389230

[7] Schmidl D Bata AM Szegedi S. Influence of PFHO eye drops on tear film thickness in patients with mild to moderate DED: a randomized controlled clinical trial. J Ocul Pharmacol Ther. 2020;36:154–61. 31895638 10.1089/jop.2019.0092

[8] Tauber J Berdy GJ Wirta DL Krösser S Vittitow JL; GOBI Study Group. NOV03 for DED associated with meibomian gland dysfunction: results of the randomized phase 3 GOBI Study. Ophthalmology. 2023;130:516–24. 36574848 10.1016/j.ophtha.2022.12.021

[9] Baudouin C Messmer EM Aragona P. Revisiting the vicious circle of DED: a focus on the pathophysiology of meibomian gland dysfunction. Br J Ophthalmol. 2016;100:300–6. 26781133 10.1136/bjophthalmol-2015-307415 PMC4789719

[10] Wang Y Carreno-Galeano JT Singh RB Dana R Yin J. Long-term outcome of punctal cauterization in the management of ocular surface diseases. Cornea. 2021;40:168–71. 32467449 10.1097/ICO.0000000000002384 PMC7704919

[11] Mohamed HB Abd El-Hamid BN Fathalla D Fouad EA. Current trends in pharmaceutical treatment of DED: a review. Eur J Pharm Sci. 2022;175:106206. 35568107 10.1016/j.ejps.2022.106206

[12] Chiambaretta F Doan S Labetoulle M. A randomized, controlled study of the efficacy and safety of a new eyedrop formulation for moderate to severe dry eye syndrome. Eur J Ophthalmol. 2017;27:1–9. 27445067 10.5301/ejo.5000836

[13] Downie LE Ng SM Lindsley KB Akpek EK. Omega-3 and omega-6 polyunsaturated fatty acids for DED. Cochrane Database Syst Rev. 2019;12:CD011016. 31847055 10.1002/14651858.CD011016.pub2 PMC6917524

[14] Ribeiro MVMR Barbosa FT Ribeiro LEF de Sousa-Rodrigues CF Ribeiro EAN. Effectiveness of using preservative-free artificial tears versus preserved lubricants for the treatment of dry eyes: a systematic review. Arq Bras Oftalmol. 2019;82:436–45. 31508669 10.5935/0004-2749.20190097

[15] Tuan HI Chi SC Kang YN. An updated systematic review with meta-analysis of randomized trials on topical cyclosporin a for dry-eye disease. Drug Des Devel Ther. 2020;14:265–74. 10.2147/DDDT.S207743 PMC6974131 32021110

[16] Bashrahil B Taher N Alzahrani Z. The efficacy and safety of varenicline nasal spray for the management of dry eye signs: a systematic review and meta-analysis. BMC Ophthalmol. 2023;23:319. 37452334 10.1186/s12886-023-03069-y PMC10347795

[17] Chen J Qin G Li L. The combined impact of intense pulsed light combined and 3% diquafosol ophthalmic solution on evaporative dry eye: a randomized control study. Ophthalmol Ther. 2023;12:2959–71. 37589932 10.1007/s40123-023-00784-z PMC10640412

[18] Tian L Gao Z Zhu L. PFHO eye drops for DED associated with meibomian gland dysfunction in chinese patients: a randomized clinical trial. JAMA Ophthalmol. 2023;141:385–92. 36929413 10.1001/jamaophthalmol.2023.0270 PMC10020931

[19] Sheppard JD Kurata F Epitropoulos AT Krösser S Vittitow JL; MOJAVE Study Group. NOV03 for signs and symptoms of DED associated with meibomian gland dysfunction: the randomized phase 3 MOJAVE Study. Am J Ophthalmol. 2023;252:265–74. 36948372 10.1016/j.ajo.2023.03.008

[20] Tauber J Wirta DL Sall K Majmudar PA Willen D Krösser S; SEECASE study group. A randomized clinical study (SEECASE) to assess efficacy, safety, and tolerability of NOV03 for treatment of DED. Cornea. 2021;40:1132–40. 33369937 10.1097/ICO.0000000000002622 PMC8330824

[21] Cochrane Handbook for Systematic Reviews of Interventions | Cochrane Training. https://training.cochrane.org/handbook/current. Accessed October 2, 2023.

[22] Cochrane Handbook for Systematic Reviews of Interventions | Cochrane Training. https://training.cochrane.org/handbook. Accessed October 5, 2023.

[23] RevMan: Systematic review and meta-analysis tool for researchers worldwide | Cochrane RevMan. https://revman.cochrane.org/info. Accessed October 18, 2024.

[24] 1. Xiidra. Prescribing information. Novartis Pharmaceuticals; 2020. https://www.xiidra.com/globalassets/pdf/packageinserts/pharma/xiidra-prescribing-information.pdf. Accessed August 17, 2023. – Yahoo Search Results. https://search.yahoo.com/search;_ylt=AwrOo51K4Q9nsyEA8lVXNyoA;_ylc=X1MDMjc2NjY3OQRfcgMyBGZyA21jYWZlZQRmcjIDc2ItdG9wBGdwcmlkAwRuX3JzbHQDMARuX3N1Z2cDMARvcmlnaW4Dc2VhcmNoLnlhaG9vLmNvbQRwb3MDMARwcXN0cgMEcHFzdHJsAzAEcXN0cmwDMTU5BHF1ZXJ5AzEuJTA5WGlpZHJhLiUyMFByZXNjcmliaW5nJTIwaW5mb3JtYXRpb24uJTIwTm92YXJ0aXMlMjBQaGFybWFjZXV0aWNhbHMlM0IlMjAyMDIwLiUyMEFjY2Vzc2VkJTIwQXVndXN0JTIwMTclMkMlMjAyMDIzLiUyMGh0dHBzJTNBJTJGJTJGd3d3Lm5vdmFydGlzLmNvbSUyRnVzLWVuJTJGc2l0ZXMlMkZub3ZhcnRpc191cyUyRmZpbGVzJTJGeGlpZHJhLnBkZgR0X3N0bXADMTcyOTA5Mzk5NA--?p=1.%09Xiidra.+Prescribing+information.+Novartis+Pharmaceuticals%3B+2020.+Accessed+August+17%2C+2023.+https%3A%2F%2Fwww.novartis.com%2Fus-en%2Fsites%2Fnovartis_us%2Ffiles%2Fxiidra.pdf&fr=mcafee&type=E210US91215G91802&fr2=sb-top. Accessed October 16, 2024.

[25] Protzko EE Segal BA Korenfeld MS Krösser S Vittitow JL. Long-term safety and efficacy of PFHO ophthalmic solution for the treatment of patients with DED: the KALAHARI study. Cornea. 2024;43:1100–7. 37921522 10.1097/ICO.0000000000003418 PMC11296276

[26] 1. Lacrisert. Prescribing information. Bausch + Lomb; 2019. https://www.drugs.com/pro/lacrisert.html. Accessed August 17, 2023. – Yahoo Search Results. https://search.yahoo.com/search;_ylt=Awr917NE4A9nmw8DYupXNyoA;_ylc=X1MDMjc2NjY3OQRfcgMyBGZyA21jYWZlZQRmcjIDc2ItdG9wBGdwcmlkA1haT3IxajgwUVFlZUViczBzM0J3ZkEEbl9yc2x0AzAEbl9zdWdnAzEEb3JpZ2luA3NlYXJjaC55YWhvby5j b20EcG9zAzAEcHFzdHIDBHBxc3RybAMwBHFzdHJsAzE0NARxdWVyeQMxLiUyMExhY3Jpc2VydC4lMjBQcmVzY3JpYmluZyUyMGluZm9ybWF0aW9uLiUyMEJhdXNjaCUyMCUyQiUyMExvbWIlM 0IlMjAyMDE5LiUyMEFjY2Vzc2VkJTIwQXVndXN0JTIwMTclMkMlMjAyMDIzLiUyMGh0dHBzJTNBJTJGJTJGd3d3LmxhY3Jpc2VydC5jb20lMkZzaXRlYXNzZXRzJTJGcGRmJTJGTGFjcmlzZXJ0LXBhY2thZ2UEdF9zdG1wAzE3MjkwOTM3Mjc-?p=1.+Lacrisert.+Prescribing+information.+Bausch+%2B+Lomb%3B+2019.+Accessed+August+17%2C+2023.+https%3A%2F%2Fwww.lacrisert.com%2Fsiteassets%2Fpdf%2FLacrisert-package&fr=mcafee&type=E210US91215G91802&fr2=sb-top. Accessed October 16, 2024.

[27] 1. Tyrvaya. Prescribing information. Viatris; 2021. https://www.tyrvaya-pro.com/files/prescribing-information.pdf. Accessed August 17, 2023. – Yahoo Search Results. https://search.yahoo.com/search;_ylt=AwrO8M254A9nDcICchdXNyoA;_ylc=X1MDMjc2NjY3OQRfcgMyBGZyA21jYWZlZQRmcjIDc2ItdG9wBGdwcmlkA2llN2g3RlhuUW1DQXY3UU9xNDVzY0EEbl9yc2x0AzAEbl9zdWdnAzIwBG9yaWdpbgNzZWFyY2gueWFob28uY29tBHBvcwMwBHBxc3RyAwRwcXN0cmwDMARxc3RybAMxMzkEcXVlcnkDMS4lMDlUeXJ2YXlhLiUyMFByZXNjcmliaW5nJTIwaW5mb3JtYXRpb24uJTIwVmlhdHJpcyUzQiUyMDIwMjEuJTIwQWNjZXNzZWQlMjBBdWd1c3QlMjAxNyUyQyUyMDIwMjMuJTIwaHR0cHMlM0ElMkYlMkZ3d3cudHlydmF5YS1wcm8uY29tJTJGZmlsZXMlMkZwcmVzY3JpYmluZy1pbmZvcm1hdGlvbi5wZGYEdF9zdG1wAzE3MjkwOTM4NDg-?p=1.%09Tyrvaya.+Prescribing+information.+Viatris%3B+2021.+Accessed+August+17%2C+2023.+https%3A%2F%2Fwww.tyrvaya-pro.com%2Ffiles%2Fprescribing-information.pdf&fr=mcafee&type=E210US91215G91802&fr2=sb-top. Accessed October 16, 2024.

[28] 1. Restasis. Prescribing information. Allergan; 2017. https://www.rxabbvie.com/pdf/restasis_pi.pdf. Accessed August 17, 2023. – Yahoo Search Results. https://search.yahoo.com/search;_ylt=Awr9zXXZ4A9nBgIAEidXNyoA;_ylc=X1MDMjc2NjY3OQRfcgMyBGZyA21jYWZlZQRmcjIDc2ItdG9wBGdwcmlkAwRuX3JzbHQDMARuX3N1Z2cDMARvcmlnaW4Dc2VhcmNoLnlhaG9vLmNvbQRwb3MDMARwcXN0cgMEcHFzdHJsAzAEcXN0cmwDMTI0BHF1ZXJ5AzEuJTA5UmVzdGFzaXMuJTIwUHJlc2NyaWJpbmclMjBpbmZvcm1hdGlvbi4lMjBBbGxlcmdhbiUzQiUyMDIwMTcuJTIwQWNjZXNzZWQlMjBBdWd1c3QlMjAxNyUyQyUyMDIwMjMuJTIwaHR0cHMlM0ElMkYlMkZ3d3cucnhhYmJ2aWUuY29tJTJGcGRmJTJGcmVzdGFzaXNfcGkucGRmBHRfc3RtcAMxNzI5MDkzOTEz?p=1.%09Restasis.+Prescribing+information .+Allergan%3B+2017.+Accessed+August+17%2C+2023.+https%3A%2F%2Fwww.rxabbvie.com%2Fpdf%2Frestasis_pi.pdf&fr=mcafee&type=E210US91215G91802&fr2=sb-top. Accessed October 16, 2024.

[29] 1. Cequa. Prescribing information. Sun Pharmaceutical Industries Limited; 2022. https://www.cequa.com/CequaPI.pdf. Accessed August 17, 2023. – Yahoo Search Results. https://search.yahoo.com/search;_ylt=Awr9_k0Z4Q9n.gEAQpxXNyoA;_ylc=X1MDMjc2NjY3OQRfcgMyBGZyA21jYWZlZQRmcjIDc2ItdG9wBGdwcmlkA1ZuX0NHTHNmU1EyTm9ldXNCYlZ3NEEEbl9yc2x0AzAEbl9zdWdnAzIwBG9yaWdpbgNzZWFyY2gueWFob28uY29tBHBvcwMwBHBxc3RyAwRwcXN0cmwDMARxc3RybAMxMzkEcXVlcnkDMS4lMDlDZXF1YS4lMjBQcmVzY3JpYmluZyUyMGluZm9ybWF0aW9uLiUyMFN1biUyMFBoYXJtYWNldXRpY2FsJTIwSW5kdXN0cmllcyUyMExpbWl0ZWQlM0IlMjAyMDIyLiUyMEFjY2Vzc2VkJTIwQXVndXN0JTIwMTclMkMlMjAyMDIzLiUyMGh0dHBzJTNBJTJGJTJGd3d3LmNlcXVhLmNvbSUyRkNlcXVhUEkucGRmBHRfc3 RtcAMxNzI5MDkzOTQy?p=1.%09Cequa.+Prescribing+information.+Sun+Pharmaceutical+Industries+Limited%3B+2022.+Accessed+August+17%2C+2023.+https%3A%2F%2Fwww.cequa.com%2FCequaPI.pdf&fr=mcafee&type=E210US91215G91802&fr2=sb-top. Accessed October 16, 2024.

[30] 1. Vevye. Prescribing information. Novaliq; 2023. https://www.accessdata.fda.gov/drugsatfda_docs/label/2023/217469s000lbl.pdf. Accessed August 24, 2023 . – Yahoo Search Results. https://search.yahoo.com/search;_ylt=Awr48Jg24Q9nKEMDMvJXNyoA;_ylc=X1MDMjc2NjY3OQRfcgMyBGZyA21jYWZlZQRmcjIDc2ItdG9wBGdwcmlkAwRuX3JzbHQDMARuX3N1Z2cDMARvcmlnaW4Dc2VhcmNoLnlhaG9vLmNvbQRwb3MDMARwcXN0cgMEcHFzdHJsAzAEcXN0cmwDMTUxBHF1ZXJ5AzEuJTA5VmV2eWUuJTIwUHJlc2NyaWJpbmclMjBpbmZvcm1hdGlvbi4lMjBOb3ZhbGlxJTNCJTIwMjAyMy4lMjBBY2Nlc3NlZCUyMEF1Z3VzdCUyMDI0JTJDJTIwMjAyMy4lMjBodHRwcyUzQSUyRiUyRnd3dy5hY2Nlc3NkYXRhLmZkYS5nb3YlMkZkcnVnc2F0ZmRhX2RvY3MlMkZsYWJlbCUyRjIwMjMlMkYyMTc0NjlzMDAwbGJsLnBkZgR0X3N0bXADMTcyOTA5Mzk2Mg--?p=1.%09Vevye.+Prescribing+information.+Novaliq%3B+2023.+Accessed+August+24%2C+2023.+https%3A%2F%2Fwww.accessdata.fda.gov%2Fdrugsatfda_docs%2Flabel%2F2023%2F217469s000lbl.pdf&fr=mcafee&type=E210US91215G91802&fr2=sb-top. Accessed October 16, 2024.

[31] 1. Eysuvis. Prescribing information. Kala Pharmaceuticals; 2020. https://www.eysuvis-ecp.com/pdf/prescribing-information.pdf. Accessed August 17, 2023 . – Yahoo Search Results. https://search.yahoo.com/search;_ylt=AwrO__pq4Q9nb5kBsjZXNyoA;_ylc=X1MDMjc2NjY3OQRfcgMyBGZyA21jYWZlZQRmcjIDc2ItdG9wBGdwcmlkA1pZbmNYY1NrVENhTXNKSDhYNnFxREEEbl9yc2x0AzAEbl9zdWdnAzEEb3JpZ2luA3NlYXJjaC55YWhvby5 jb20EcG9zAzAEcHFzdHIDBHBxc3RybAMwBHFzdHJsAzE1MARxdWVyeQMxLiUwOUV5c3V2aXMuJTIwUHJlc2NyaWJpbmclMjBpbmZvcm1hdGlvbi4lMjBLYWxhJTIwUGhhcm1hY2V1dGljYWxzJTNCJTIwMjAyMC4lMjBBY2Nlc3NlZCUyMEF1Z3VzdCUyMDE3JTJDJTIwMjAyMy4lMjBodHRwcyUzQSUyRiUyRnd3dy5leXN1dmlzLWVjcC5jb20lMkZwZGYlMkZwcmVzY3JpYmluZy1pbmZvcm1hdGlvbi5wZGYEdF9zdG1wAzE3MjkwOTQwMTE-?p=1.%09Eysuvis.+Prescribing+information.+Kala+Pharmaceuticals%3B+2020.+Accessed+August+17%2C+2023.+https%3A%2F%2Fwww.eysuvis-ecp.com%2Fpdf%2Fprescribing-information.pdf&fr=mcafee&type=E210US91215G91802&fr2=sb-top. Accessed October 16, 2024.

[32] Sheppard JD Evans DG Protzko EE. A review of the first anti-evaporative prescription treatment for DED: PFHO ophthalmic solution. Am J Manag Care. 2023;29(14 Suppl):S251–9. 37930231 10.37765/ajmc.2023.89464

[33] de Paiva CS Pflugfelder SC Ng SM Akpek EK. Topical cyclosporine a therapy for dry eye syndrome. Cochrane Database Syst Rev. 2019;2019. 10.1002/14651858.CD010051.pub2 PMC6743670 31517988

[34] Hu J Zhu S Liu X. Efficacy and safety of a vectored thermal pulsation system (Lipiflow®) in the treatment of meibomian gland dysfunction: a systematic review and meta-analysis. Graefe's Arch Clin Exp Ophthalmol = Albrecht von Graefes Archiv fur klinische und experimentelle Ophthalmologie. 2022;260:25–39. 34374808 10.1007/s00417-021-05363-1

